# Design and Evaluation of Korean Electropalatography (K-EPG)

**DOI:** 10.3390/s21113802

**Published:** 2021-05-31

**Authors:** Seong-Tak Woo, Ji-Wan Ha, Sungdae Na, Hyunjoo Choi, Sung-Bom Pyun

**Affiliations:** 1 Department of Electronic Engineering, Dong Seoul University, 76 Bokjeong-ro, Sujeong-gu, Seongnam-si 13117, Korea; 2 Department of Speech Pathology, Daegu University, 201, Daegudae-ro, Gyeongsan-si 38453, Korea; jw-ha@daegu.ac.kr; 3 Department of Biomedical Engineering, Kyungpook National Universicy Hospital, 130 Dongdeok-ro, Jung-gu, Daegu-si 41944, Korea; bluepoison14@gmail.com; 4 Department of Communication Disorders, Korean Nazarene University, Wolbong-ro 48, Cheonan-si 31172, Korea; hjchoi@kornu.ac.kr; 5 Department of Physical Medicine and Rehabilitation, Korea University College of Medicine, 73 Goryeodae-ro, Seongbuk-gu, Seoul 02841, Korea; rmpyun@korea.ac.kr

**Keywords:** tongue location detection, articulation disorders, tongue contact force, speech sound disorders, dysarthria

## Abstract

Recently, the development of medical rehabilitation technology has resulted in an increased interest in speech therapy equipment. In particular, research on articulation therapy for communication disorders is being actively conducted. The existing methods for the diagnosis and treatment of speech disorders, such as traditional tactile perception tests and methods based on the empirical judgment of speech therapists, have many limitations. Moreover, the position and contact force of the tongue are key factors in speech disorders with regards to articulation. This is a very important factor in the distinction of Korean characters such as lax, tense and aspirated consonants. In this study, we proposed a Korean-electropalatography (EPG) system to easily measure and monitor the position and contact force of the tongue during articulation treatment and diagnosis. In our proposed K-EPG system, a sensor was fabricated using an AgCl electrode and biocompatible silicon. Furthermore, the measured signal was analyzed by implementing a bio-signal processing module and monitoring program. In particular, the bio-signal was measured by inserting the device into the palate of an experimental healthy test group (four subjects). Through these experiments, we confirmed that our K-EPG system could be applied to clinical treatment in speech therapy.

## 1. Introduction

Recent developments in medical technology have increased the interest in rehabilitation devices. Among them, diagnostic technologies and devices related to speech therapy are continuously being studied [1,2,3,4,5,6,7]. Speech sound disorder is the most common childhood communication disorder, and articulation is very closely related to speech sound disorder [8]. Even after school age, the problem persists, resulting in residual errors that continue to create pronunciation problems even in adulthood [9,10]. This residual error means that the target phoneme has been reached but is pronounced in a distorted fashion, such as pronouncing [s] as [θ]. If the issue is severe, the unclear pronunciation causes problems such as communication disorders, difficulties, and decreased quality of life. The diagnosis and treatment of speech sound disorders, including traditional tactile perception tests and methods based on the subjective judgment of speech-based speech therapists, have many limitations [5]. In articulation movement, which is a key factor in speech sound disorder, the position and tension of the tongue (i.e., lax, tense and aspirated consonants) are very important factors [11,12,13]. Furthermore, some researchers note in their studies the effect of temporomandibular disorders on speech. This study suggests that the correct position of the tongue for each phoneme in the oral cavity has a greater impact on speech sound disorders than on the masticatory system or temporomandibular disorders [14].

Some researchers have developed devices which provide information about the movement of the tongue. However, these techniques still show limitations in the location and the contact force of the tongue for Korean characteristics. There are various devices for use in speech sound disorders; of these, the electropalatography system provides information about the location of the tongue and the timing during contact. Analyzing individual pronunciation characteristics is easy by obtaining information about the position and timing of the tongue for each phoneme in the oral cavity. However, information about the contact force acting on each phoneme is insufficient [15,16,17]. In addition, a tongue interface module based on a magneto-inductive sensor can be viewed as a device for convenience in life, similar to a controllable wheelchair for the physically handicapped, rather than a speech sound disorder device [18]. The segmental vocoder is another communication device for laryngectomized patients. This technology captures the movement of the lips and tongue using ultrasound and optical image measurements, and then synthesizes sentences for the patient to communicate. However, information about the contact force of the tongue is still insufficient [19]. In particular, the contact force of the tongue for each alveolum, palato-alveolum, and velum phoneme is a crucial factor in speaking. In Korean, the distinction between lax, tense and aspirated consonants is clear, with each having its own distinct phonology, which is very important because the meanings of the words are distinct.

**Figure 1:**
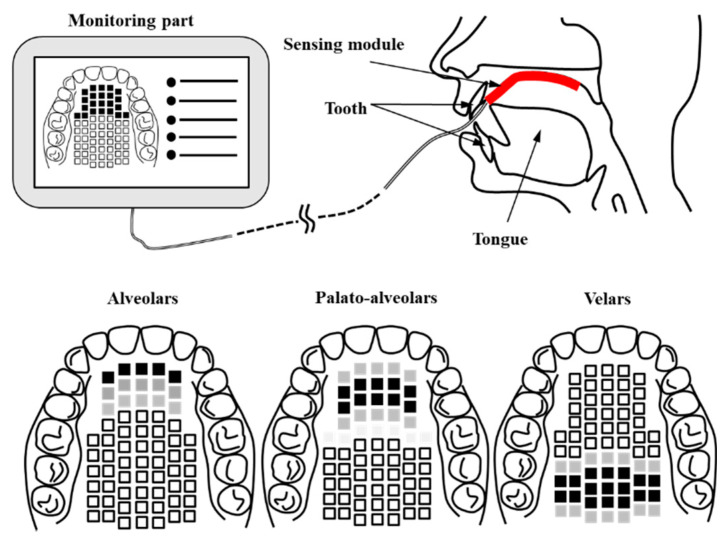
Schematic of the proposed Korean-electropalatography (EPG) system and the position of the tongue.

In this work, we propose an AgCl electrode-based Korean electronic palatal sensor structure to easily measure and monitor the location and contact force of the tongue in articulation treatment and diagnosis. In addition, a signal processing module was implemented and combined with the sensor unit in order to evaluate the healthy control group. Figure 1 shows a conceptual diagram of the proposed K-EPG (Korean electropalatography) sensor and module. The proposed K-EPG sensor is able to distinguish the position and contact force based on the contact of the tongue with the AgCl electrode.

Additionally, the biocompatible material was coated with silicone to enable temporary application in the body. We implemented a signal processing module to distinguish the rigidity by measuring the intensity of the biosignal based on the contact of the tongue. The monitoring unit provides information to the therapist and the patient in real time.

As shown in Figure 1, the K-EPG sensor is attached from the alveolo-palatal to the velar-palatal region. The data is transmitted to the monitoring unit through the signal processing module whenever the tongue comes into contact with the K-EPG attached to the upper part of the oral cavity. The Korean language is divided into ‘lax’, ‘tense’, and ‘aspirated’ consonants based on the contact force of the tongue. A lax consonant is a consonant with relatively weak tongue contact and causing lower oral pressure than that in tense or aspirated consonants.

Tense and aspirated consonants have stronger tongue contact and higher oral pressure than those for lax consonants. The oral pressure is not higher because of a decrease in the volume of the oral cavity, but rather an increase in pressure caused by an increase in expiratory strength while pronouncing tense or aspirated consonants. Table 1 shows the classification of Korean consonants based on the tongue position and intensity of production. The marked Korean consonants can be displayed according to the international phonetic alphabet (IPA). In this paper, the location and contact force of the tongue were analyzed by the AgCl electrode-based K-EPG sensor and signal processing module. Through healthy control group evaluation, it was confirmed that our sensor can be applied in clinical practice for actual therapy for speech sound disorder.

## 2. K-EPG System

### 2.1. Design of the K-EPG Sensor

**Figure 2:**
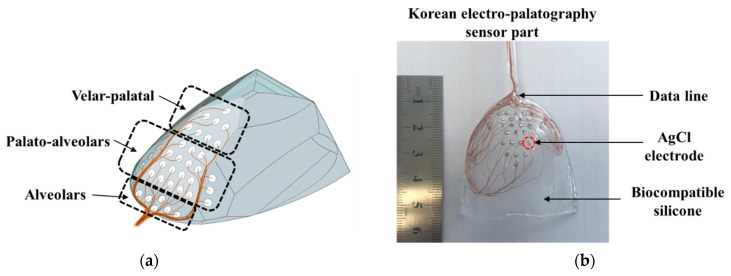
(**a**) The schematic and (**b**) a picture of the fabricated Korean-electropalatography sensor.

The electrode position is an important factor in the detection of tongue information. In particular, during verbalization, the most contact between the tongue and palate occurs in the alveolum region. In this paper, the K-EPG sensor was fabricated using 36 arranged AgCl electrodes with diameters of 1.5 mm each that were coated with biocompatible silicon (C6-560, C6-540, Dow Corning corporation). The fabricated K-EPG sensor and conceptual diagram are shown in Figure 2.

In total, 28 electrodes were placed in the hard palate region (of these, 36 electrodes were placed in the alveolum and palato-alveolum regions), and 8 electrodes were placed in the velar region. In particular, the electrodes were focused on the alveolar and the palato-alveolar regions, which are the main areas with which the tip of the tongue comes into contact during verbalization. The K-EPG sensor unit should be in close contact with the palato-alveolar area in the oral cavity in order to minimize discomfort during verbalization. Therefore, a palatal model was prepared using a dental impression material (Peak light body, Neosil) and then 3D scanning was performed in order to fabricate the EPG sensor section. Then, biocompatible silicone C6-560 part A and C6-540 part B were blended 1:1 and inserted into a mold on which the electrodes were placed. All 36 silicone-coated electrodes (the sensor part of the K-EPG system) were dried at 100 °C for 3 h. The K-EPG sensor was manufactured with a length of 55 mm, a width of 45 mm and a thickness of 1.5 mm. Each AgCl electrode was inserted to protrude about 0.2 mm for easy contact with the tongue.

### 2.2. Bio-Signal Processing Technology

**Figure 3:**
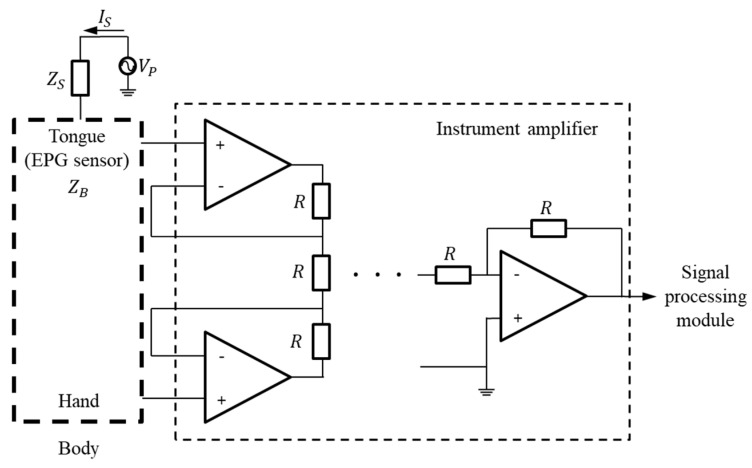
Block diagram for the measurement of signals based on tongue contact.

In general, the potential on the skin surface is generated by ions passing through the cell membranes of neurons [20,21]. Because these nerve tissues are surrounded by a conductive medium, the generated electric current is conducted up to the skin’s surface, and the electric potential appears by Ohm’s law. The proposed K-EPG system is a method that uses an electrode to measure an induced electric signal, thereby detecting the electrical processes of the human body. Sometimes, even if the tongue is not in contact with the K-EPG sensor that is inserted into the oral cavity, some electrical noise may be caused by the silicone attached to the ceiling of the oral cavity. Therefore, by implementing a differential amplifier in the K-EPG system, all of the measured signals are considered noise and adjusted, even when the tongue is not in contact with the electrode. When the tongue comes into contact with the electrode on the sensor, the device measures a strong electrical signal with a magnitude corresponding to the strength of the contact force. Studies that have used the existing electropalatography system confirmed many electrode activations mainly in the alveolum and palato-alveolum regions. In this study, the electrode activation was approximately 2.5 times higher in/t/consonants than in/k/consonants. Moreover, these results were confirmed in test groups between the ages of 6 to 38 years [16]. The proposed K-EPG sensor was configured with 36 electrodes, of which 28 were placed in the alveolum and palato-alveolum areas, and the remaining eight in the velum area. The signal measurement structure is shown in Figure 3.

If the potential on the tongue surface is called *V_p_*, the basic impedance of the AgCl electrode in the EPG sensor is *Z_B_*, and the impedance change due to tongue contact is Δ*Z*, then the electrode impedance *Z_R_* is as shown in Equation (1) below. In addition, if the basic impedance of the human body is *Z_S_*, and the microcurrent flowing through the surface of the tongue is *I_S_*, then the voltage *V_ZR_* at the electrode can be expressed as in Equation (2).

(1) $$Z_{R}=Z_{B}+\Delta Z$$

(2) $$V_{Z_{R}}=I_{S}\times (Z_{S}+Z_{B}+\Delta Z)$$

The biosignal exhibits a constant bias in its voltage level due to the body impedance *Z_S_* and the electrode impedance *Z_R_*. Additionally, the measured biosignal is proportional to the impedance change Δ*Z* between the tongue and the AgCl electrode. The bias voltage level, *V_BL_*, is expressed by Equation (3) below. In fact, the bias voltage level changes in impedance based on physiological factors. Therefore, in this paper, as shown in Figure 3, the bias voltage is removed by connecting other contacts in the human body. Then, the biosignal can be obtained by differentially amplifying the amount of change according to the contact intensity of the tongue. The signal change, Δ*V*, of the electrode according to the contact of the tongue can be expressed by Equation (4).

(3) $$V_{BL}=I_{S}\times (Z_{S}+Z_{B})$$

(4) $$\Delta V=V_{Z_{R}}-V_{BL}=I_{S}\times \Delta Z$$

### 2.3. Fabrication of the K-EPG System

**Figure 4:**
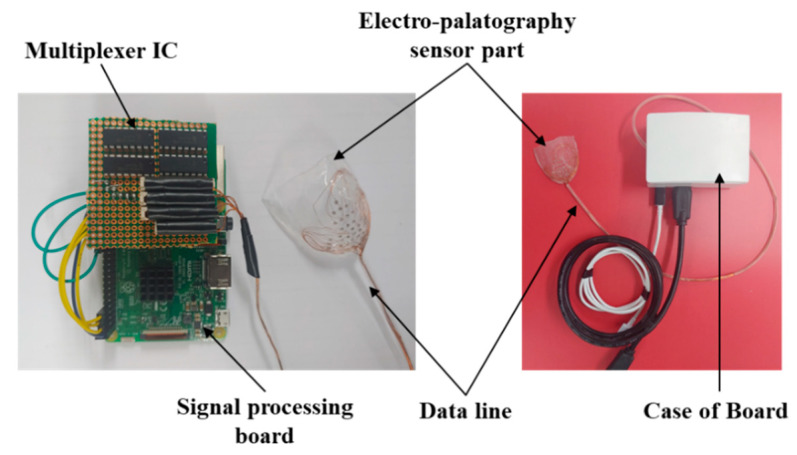
Picture of the fabricated signal processing module and the electropalatography sensor.

The EPG system was implemented by combining the coated sensor with the biocompatible material and the signal processing board, as shown in Figure 4. The AgCl electrode measures the biosignal based on tongue contact, and the signal is transmitted to the signal processing module based on a universal development board (Raspberry pi3 B+, Allied electronics Inc., Chicago, IL, USA) to transmit information to the monitoring unit.

**Figure 5:**
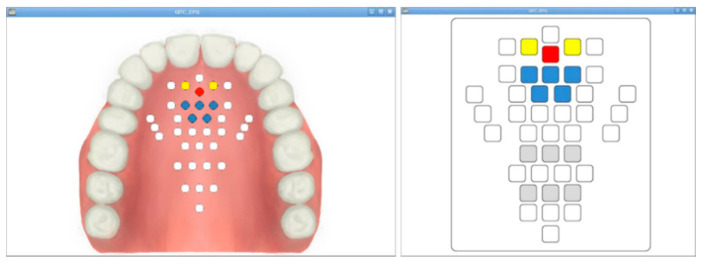
The fabricated monitoring program: the anatomical type and classic type.

The signal measured by the 36 AgCl electrodes is divided through a CMOS logic-based analog multiplexer IC (CD74HC4067, Texas Instruments) and transmitted to the signal processing module. In addition, the transmitted analog signal was set to ADC (analog to digital convert) with a 10 bit resolution, and a PC-based graphical user interface (GUI) was implemented to monitor the tongue position and contact force level information. The monitoring program is shown in Figure 5. The monitoring program was developed through the C++ based Qt Library in the Linux based Ubuntu OS (operating system) environment. In addition, in order to visualize the ADC data in real time, it was processed through DSP (Digital Signal Processing), and a GUI program was implemented to monitor the image information.

### 2.4. Verification of the K-EPG Sensor

**Figure 6:**
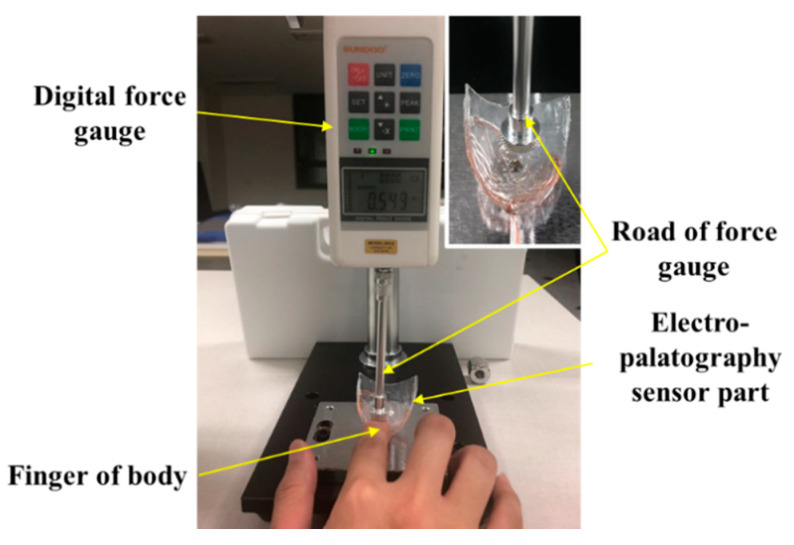
Experimental setup for the evaluation of the electropalatography sensor according to the force application.

The output according to the applied force was measured using a digital force meter (Model SH-2, Sundoo) to evaluate the characteristics of the implemented K-EPG sensor. The experimental setup is shown in Figure 6.

The AgCl electrode in the fabricated electropalatography sensor measures bio signals through tongue contact. By placing a finger between the end of the force meter and the K-EPG sensor, the output characteristics of the K-EPG sensor according to the downward force applied were evaluated. These experiments evaluated the characteristics of the electrodes in the K-EPG sensor under the assumption that the impedances in the human body were similar, as in Equations (1)–(4) mentioned above. In addition, the fixed bias voltage was removed by connecting other parts (hands) of the human body to the channel of the differential amplifier, and only the biosignal change was measured. In general, when the tongue comes into contact with the palatal area, the contact force of the tongue is about 0.01 to 0.1 N. Some researchers have analyzed the contact force of the tongue during normal and deglutition movements using flexible force sensors, with the force ranging from 0.01 to 1.67 N [22]. In order to classify the contact force levels of the tongue, we applied a force of about 0.01 to 2.0 N by pressing the K-EPG sensor with a finger, as shown in Figure 6. The output voltage characteristics measured from the sensor were analyzed. For contact force ranging from 0.02 to 0.1 N, the measurements were performed at intervals of 0.02 N, whereas between 0.1 N to 2.0 N, 10 measurements were performed at a 0.1 N interval.

**Figure 7:**
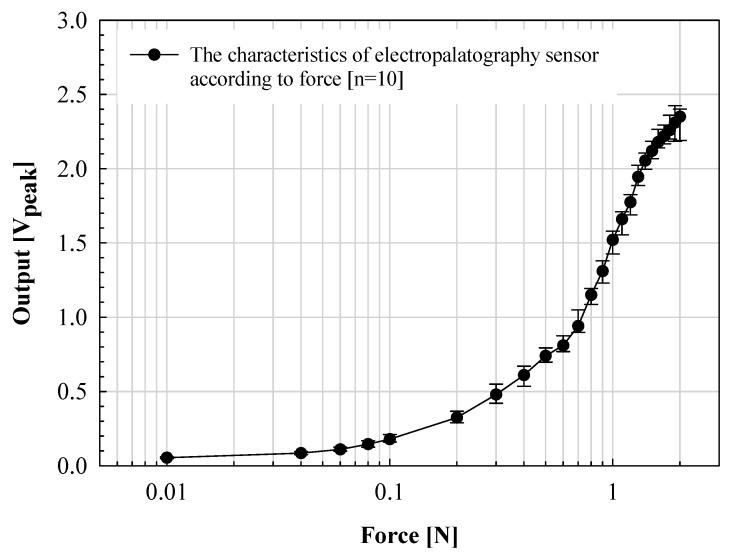
Verification of the proposed electropalatography sensor according to the force application.

While speaking, the tongue contact force is relatively low, less than 0.1 N. However, because deglutition movement is also an important factor in the evaluation of articulation motion, in this paper, the output characteristics according to the applied force in the range of 0.01 to 2.0 N were evaluated. The results are shown in Figure 7.

The manufactured K-EPG sensor was measured within a standard deviation of ±0.15 V from 0.02 to 1.0 N. The voltage output linearly increased with the applied force. However, a standard deviation of up to ±0.35 V was measured in the above sections. In particular, for forces above 1.6 N, the increase width was significantly lowered. This is because when the applied force increases above a certain level, the voltage measured through contact with the finger converges to the bias voltage level. The electrical signal (analog voltage) measured through the electrode was classified by contact force through analog to digital conversion using the signal processing board. The signals were expressed by classifying the signals measured from 0 to 0.1 V in the low section, 0.1 to 0.2 V in the middle section, and 0.2 V or higher in the high section. Each of these voltages corresponds to a contact force (applied indirectly through the finger) in the range of 0 to 0.04 N, 0.04 to 0.08 N and 0.08 N or more for the low, middle and high sections, respectively. The position and intensity information for each phoneme transmitted through the signal processing board is expressed in the implemented GUI.

## 3. Experiments and Results

### 3.1. Healthy Test Group and Experiments

**Figure 8:**
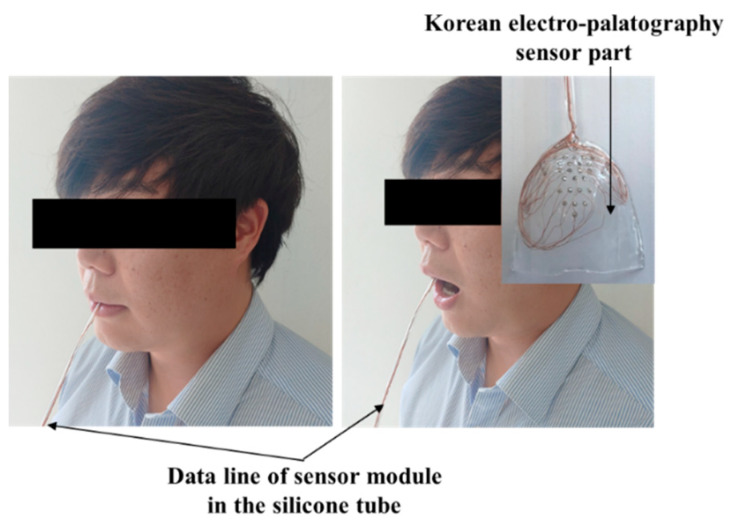
Experimental setup for the evaluation of the electropalatography system in the healthy control group (man, aged 35 years old).

The healthy test group evaluation was performed using the K-EPG system. This study was conducted with prior approval from the Institutional Review Board (IRB, No. 1040621-201907-HR-061-02). The healthy control group consisted of two males (ages 31 and 35) and two females (ages 35 and 36). The healthy test group contained people who did not have underlying speech-related disorders. All of the participants volunteered and did not receive any financial support. The personal information of the participants is protected by the management of the Kyungil University Bioethics Committee. The participants were asked to speak nine syllables, and the position and contact force of tongue were measured using a K-EPG sensor inserted into the oral cavity. The following nine syllables, including consonants and vowels corresponding to the alveolar, palato-alveolar, and velar regions were used in the experiment: /ʈa/; /ʈ^*^a/; /ʈ^h^a/; /ʨa/; /ʨ^*^a/; /ʨ^h^a/; /ka/; /k^*^a/; and/k^h^a/. The healthy control group experiment was conducted in the presence of a speech therapist and with the informed consent of the subjects. A K-EPG sensor was inserted into the subject’s mouth, and the sensor was attached and fixed to the tip of the palate using dental cement (PEAK, Neosil). The experimental setup is shown in Figure 8.

### 3.2. Results

**Figure 9:**
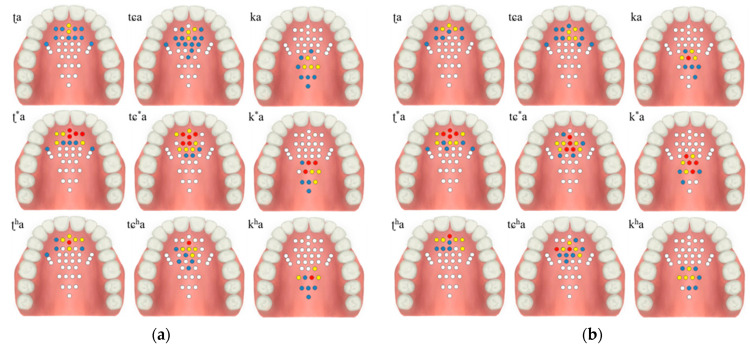

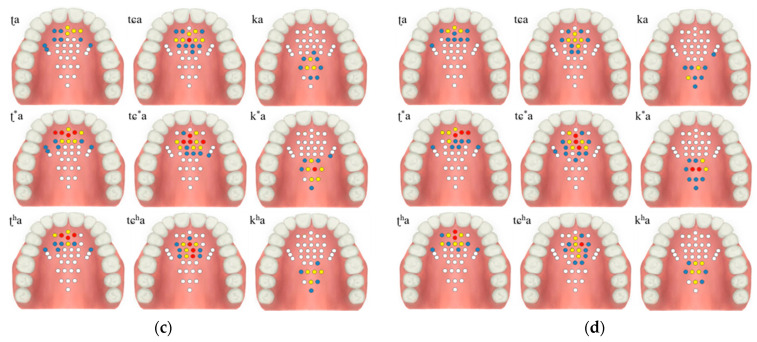
Electropalatography results from the experimental control group: (**a**) male 1, (**b**) male 2, (**c**) female 1, and (**d**) female 2.

The contact force level based on the tongue stiffness measured in the monitoring unit is expressed in red (strong), yellow (medium), blue (weak), and white (non-contact). The electrodes’ activation characteristics are shown in Table 2 and Figure 9, below. During the experiment, more electrodes were activated in the lax than in the aspirated or tense consonants in both men and women.

The tongue contact force decreased in the order of tense, aspirated and lax consonants, with similar characteristics in both men and woman. In particular, the aspirated sound in the form of pronouncing a long sound in the oral cavity was measured to have a relatively low contact force compared to the tense sound.

## 4. Discussion and Conclusions

In this work, we implemented a Korean electropalatography system that can be easily inserted into the oral cavity for articulation therapy for people with communication disorders. In particular, an experiment was conducted on subjects using Korean-based consonants and vowels. In addition, information on the contact position and contact force of the tongue for each phoneme was confirmed through the experimental results. These experimental results show that the implemented K-EPG system can be effectively used for articulation therapy.

The implemented K-EPG sensor can be easily manufactured using AgCl electrodes and biocompatible silicon, which are commonly used for the measurement of biometric signals. We arranged these electrodes after considering the contact position of the tongue during lax, tense and aspirated consonant sounds. The proposed K-EPG sensor was classified into three types by contact strength according to the characteristics of Korean consonant strength. This method can be analyzed objectively in articulation therapy, so improvements in treatment are expected.

In addition, multi-electrode analog signal collection and bio-signal processing functions were implemented using a signal processing board, and a PC-based electronic palate monitoring program was implemented to visually show the measurement results to the user. Load application evaluation based on the force meter was performed, and the sensitivity of the K-EPG sensor was measured. We confirmed that the contact force of tongue can be effectively measured in the range of 0.01 to 0.1 N. The fabricated K-EPG system is capable of detecting the position and contact force of the tongue, but additional research is still required.

In order to standardize the position of the proposed electrode, it is essential to analyze the experimental results under the same conditions with various electrode arrangements in future research. Cheng et al. [16] analyzed the tongue patterns for consonants in participants aged 6 to 38 years using an electropalatography system, wherein they tried to identify and standardize the differences in tongue patterns based on age and speech disorders. Furthermore, the activation characteristics of the electrodes were studied by placing 62 electrodes equally in the alveolum and velum areas. As expected, high electrode activity was confirmed in the alveolum region. Most of the studies that have used electropalatography focused on the position of the tongue and the maximum contact pattern based on repetitive learning. This pattern analysis is very important for the electropalatography system to provide visual feedback to the patient and bring about improvement through the rehabilitation [15,16,17,23,24,25]. The proposed K-EPG system also requires additional iterations to enhance the sensor’s sensitivity and maximize the contact pattern in order to more accurately determine the contact force level. 

In this study, we divided 36 electrodes among the alveolar, palato-alveolar and velar regions to match Korean words. The experimental results indicate that the electrode activity was high in the alveolar and palato-alveolar regions. However, our work requires additional experimentation to optimize the electrode placement. In particular, it is necessary to consider the position and contact force of tongue at the back of the palate by placing an electrode on the inner tooth side of the palate. Our current focus was on the position and contact force of tongue in the front of the palate. Moreover, our study only reported results for four healthy participants; however, results from participants of all age groups are required for future studies. It is important to analyze the results of participants with sound speech disorder (SSD) [26,27,28] caused by unilateral cleft lip, Down syndrome, and Dysarthria because, ultimately, the K-EPG system is a technology for these patients. In the future, we can improve our K-EPG system based on the results of various other cases.

As shown in Table 2, the healthy participants comprised two men and two women, who did not show any differences in the position of the tongue based on gender. However, given that the number of participants was too small and that they were healthy adults, it is difficult to draw conclusions about the gender characteristics. Notably, the tense consonants had a higher level of tongue contact than that of the aspirated consonants, which was the same for both men and women. In Korean, the results of the experiment are very important because lax, tense and aspirated consonants must be clearly distinguished. Naturally, even if the sound is similar, the phonology changes based on the contact force of the tongue. In the case of Korean, the distinction between lax, tense, and aspirated consonants is clear, with each having its own distinct phonology. Therefore, it is very important to distinguish these consonants based on the contact force of the tongue, and thereby distinguish the meaning of the word.

The ultimate purpose of the K-EPG system is to be used to assist users’ judgment to-ward a more objective treatment method based on the subjective and skilled experience of speech therapists, in line with rapid advancements in rehabilitation medical technology. Therefore, by the optimization and performance of clinical trials on the K-EPG sensor, we expect that our proposed K-EPG sensor will improve speech therapy treatments.

## Figures and Tables

**Table 1:** Classification of Korean consonants based on the tongue position and the intensity of production.

		Alveolum	Palato-Alveolum	Velum
Plosive	Lax	ʈ		k
	Tense	ʈ^*^		k^*^
	Aspirated	ʈ^h^		k^h^
Fricative	Lax	s		
	Tense	s^*^		
Affricative	Lax		ʨ	
	Tense		ʨ^*^	
	Aspirated		ʨ^h^	

**Table 2:** Results of the healthy test group according to the tongue position and intensity of production.

Syllable	Desired Position of Tongue	Number of Active Electrodes				Red/Yellow/Blue			
		M1	M2	W1	W2	M1	M2	W1	W2
ʈa	Alveolum	12	10	12	11	0/2/10	0/3/7	0/4/8	0/3/8
ʈ^*^a	Alveolum	13	14	15	14	4/4/5	4/5/5	4/5/6	3/4/7
ʈ^h^a	Alveolum	11	10	12	13	1/5/4	2/5/3	3/3/6	2/5/6
ʨa	Palato-alveolum	16	14	15	12	0/3/13	0/4/10	1/6/8	0/5/7
ʨ^*^a	Palato-alveolum	14	13	16	15	5/6/3	4/5/4	4/6/6	3/5/9
ʨ^h^a	Palato-alveolum	10	12	13	14	1/4/5	2/4/6	3/4/6	1/5/8
ka	Velum	11	8	10	9	0/4/7	1/3/5	0/3/7	0/2/7
k^*^a	Velum	10	10	11	11	3/3/4	4/3/3	1/5/5	2/2/7
k^h^a	Velum	9	9	8	9	1/3/5	0/4/5	0/4/4	0/5/4
